# Traumatic Rare Volar Distal Radioulnar Joint (DRUJ) Dislocation in a Geriatric Female Patient: A Case Report

**DOI:** 10.7759/cureus.107544

**Published:** 2026-04-22

**Authors:** Felix Barth, Martin Waldner, Mathias Häfeli

**Affiliations:** 1 Cardiology, Hochgebirgsklinik Davos, Davos, CHE; 2 Internal Medicine, Landesspital Liechtenstein, Vaduz, LIE; 3 Surgery, Landesspital Liechtenstein, Vaduz, LIE; 4 General Practice, Hausarztpraxis am Bahnhof GmbH, Flums, CHE; 5 Hand Surgery, Kantonsspital Graubünden, Chur, CHE

**Keywords:** case report, closed reduction, distal radioulnar joint dislocation, druj dislocation, elderly female, volar dislocation, volar druj dislocation

## Abstract

Isolated volar distal radioulnar joint (DRUJ) dislocation is a rare type of traumatic joint dislocation and is rarely seen even by orthopaedic specialists. Surgical intervention is often not warranted, as closed reduction followed by immobilisation is often successful. This is the case of an 88-year-old woman who presented with pain in the right forearm after a fall. The diagnosis of a volar DRUJ dislocation was confirmed by radiographic imaging and treated by closed reduction. After immobilisation in a long-arm cast for three weeks and a forearm splint for another two weeks, full functionality of the hand and wrist was eventually gained after six months. The triangular fibrocartilage complex (TFCC) usually provides sufficient stability and healing to stabilise the DRUJ.

## Introduction

Dislocation of the distal radioulnar joint (DRUJ) is a rare injury, of which volar dislocation is even more uncommon than the dorsal. Forearm fractures are often associated with this type of injury; however, it was not the case in this patient. The diagnosis is usually confirmed by plain radiographs or a CT scan, although it can easily be missed as changes can be subtle. Therapy is either closed or open reduction, followed by immobilisation. There are only very few cases of volar DRUJ dislocation without fracture reported in the literature. In this case report, we aim to provide a brief overview of the literature and the biomechanics of DRUJ. We want to add further data to research to better understand, diagnose and treat this rare entity.

This case report has been reported in line with the SCARE 2020 criteria [1]. The patient gave informed verbal and written consent to publish her case and accompanying images in any medical journal.

## Case presentation

An 88-year-old woman had a fall on her dominant right hand while she was hospitalised for an acute gastroenteritis in the department of internal medicine. She was walking during the night with her mobile intravenous (IV) pole when she suddenly lost balance and slipped. The exact mechanism of injury cannot be remembered by the patient. After the fall, she complained of acute pain in her right wrist. Upon primary examination, mild swelling and tenderness to palpation over the wrist joint were noted. Supination and pronation caused extreme pain and distress in the distal forearm of the patient. No open wounds or lacerations of the skin were present, and no clinical evidence of neurovascular compromise was present. The wrist and forearm were immobilised by a structural aluminium malleable (SAM) splint until the next morning, when the initial plain radiographs were obtained.

**Figure 1:**
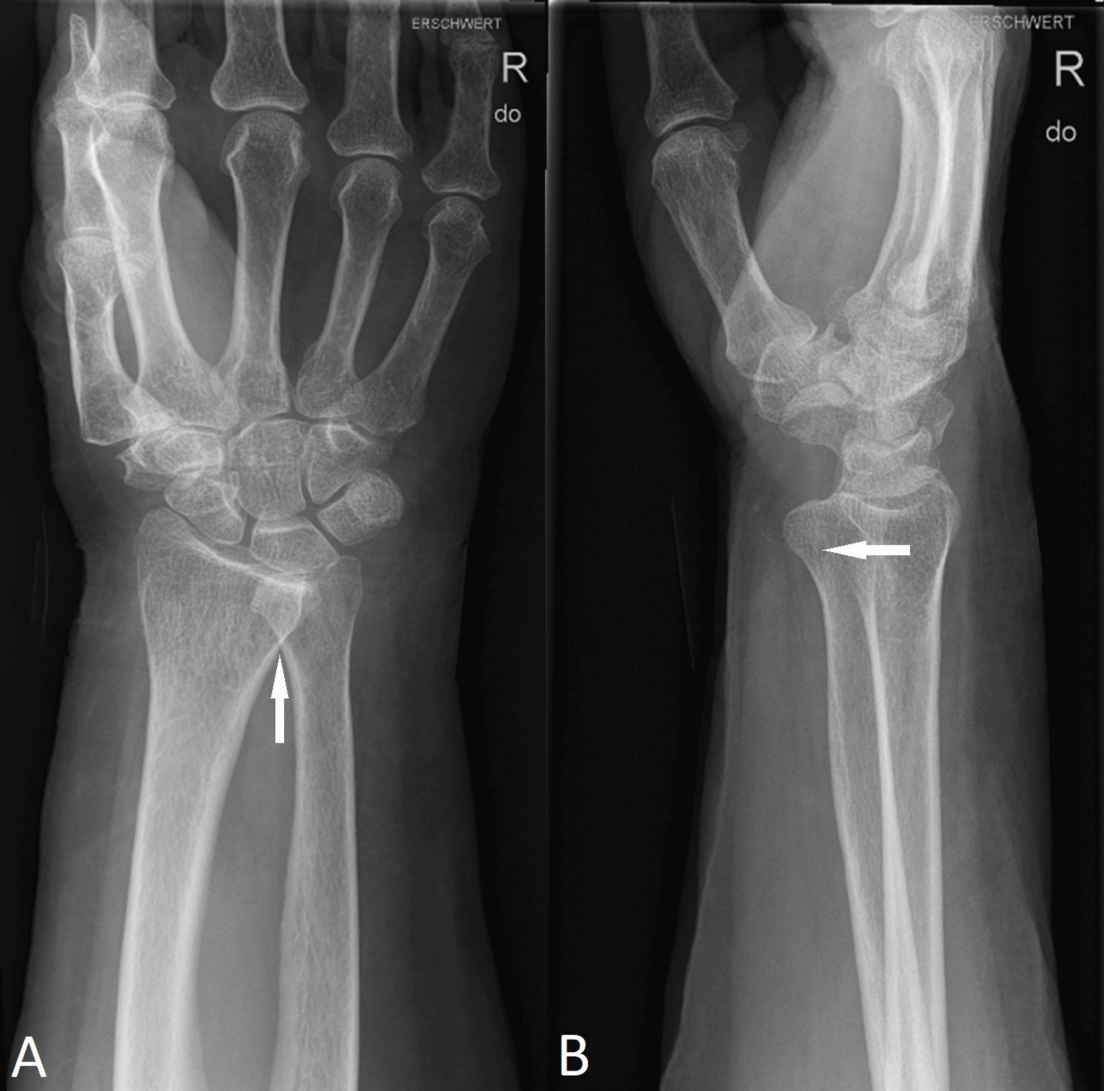
Radiographs of the right wrist showing an overlap of the distal radius and ulna (white arrow) in the posteroanterior view (A) and volar displacement of the ulnar head (white arrow) in the lateral view (B).

The posteroanterior (PA)-view plain X-ray showed overlap of the distal radial and ulnar bone (Figure 1A). In the lateral view, a volar displacement by >1 cm of the ulnar head compared to the radius was observed, and a volar DRUJ dislocation was suspected (Figure 1B).

**Figure 2:**
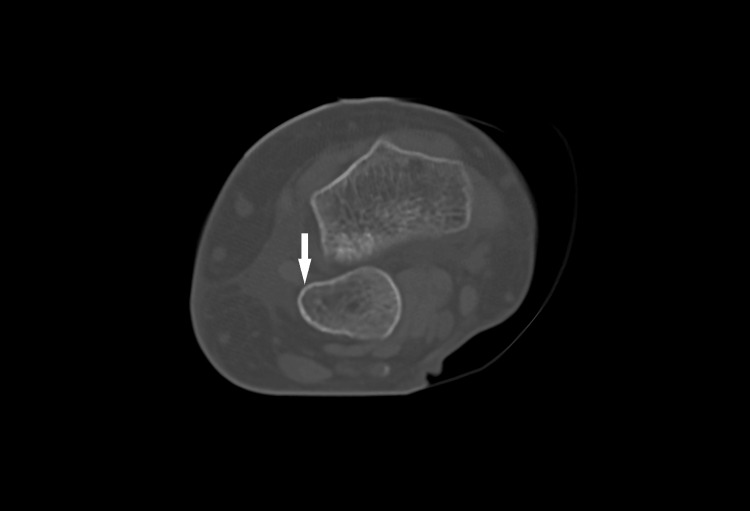
Axial plane CT scan of the right wrist showing volar (down) displacement of the ulna (white arrow) in relation to the radius. CT: computed tomography

**Figure 3:**
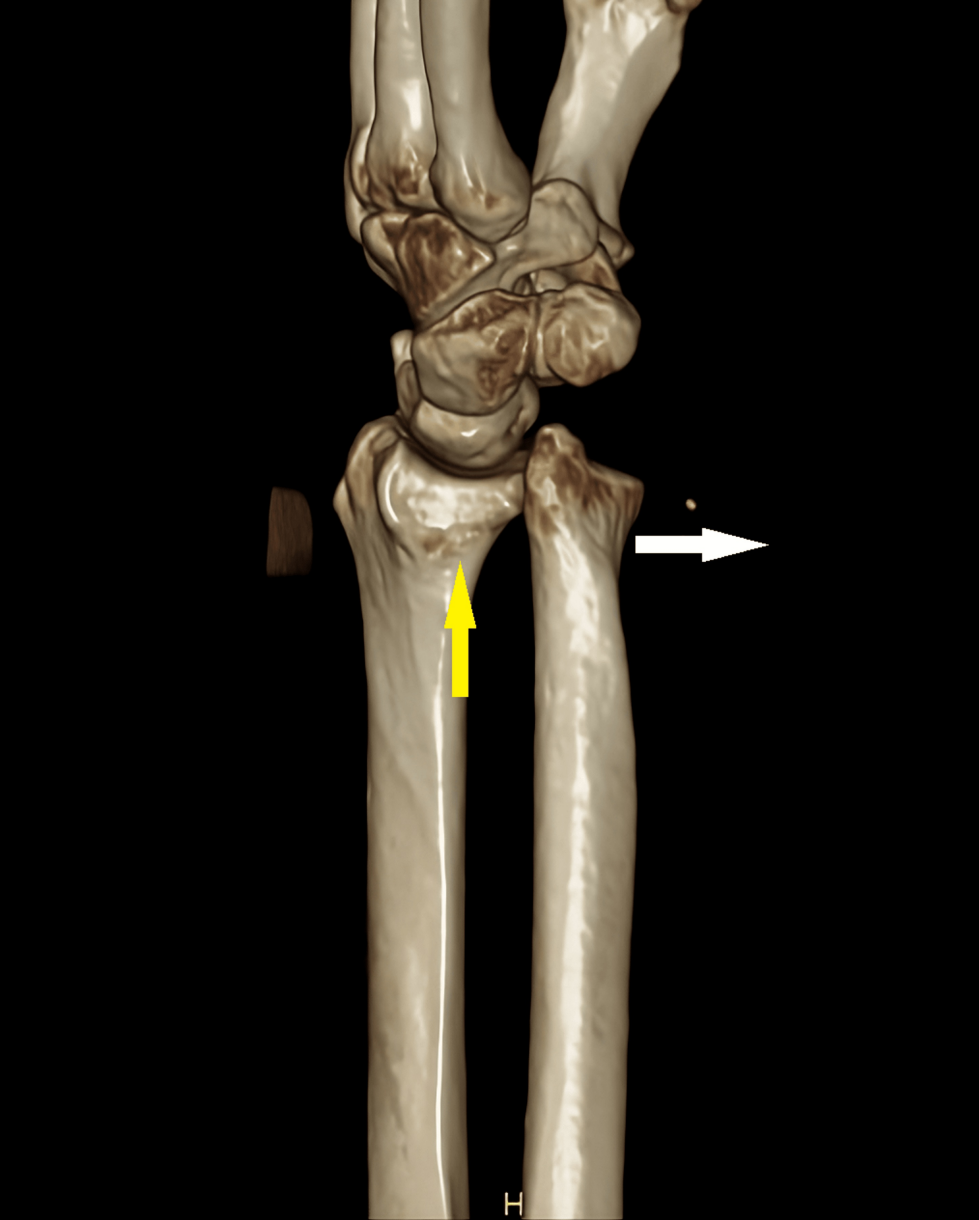
CT scan with 3D reconstruction of the right wrist showing the ulna displaced volarly (direction of white arrow) and an empty sigmoid notch of the radius (yellow arrow). CT: computed tomography, 3D: three-dimensional

For further assurance, a CT scan was performed, confirming the diagnosis of a DRUJ dislocation, where the ulna was displaced to the volar side. The radius showed signs of slight contusion, joint effusion and soft tissue oedema. There were no associated fractures of the wrist or carpal bones and no evidence of injury to the ulnar nerve (Figures 2, 3).

The volar displacement was successfully reduced under regional anaesthesia of the brachial plexus. The reposition was achieved by applying axial tension on the ulna and radial abduction of the wrist while delivering dorsally directed pressure on the ulnar head.

**Figure 4:**
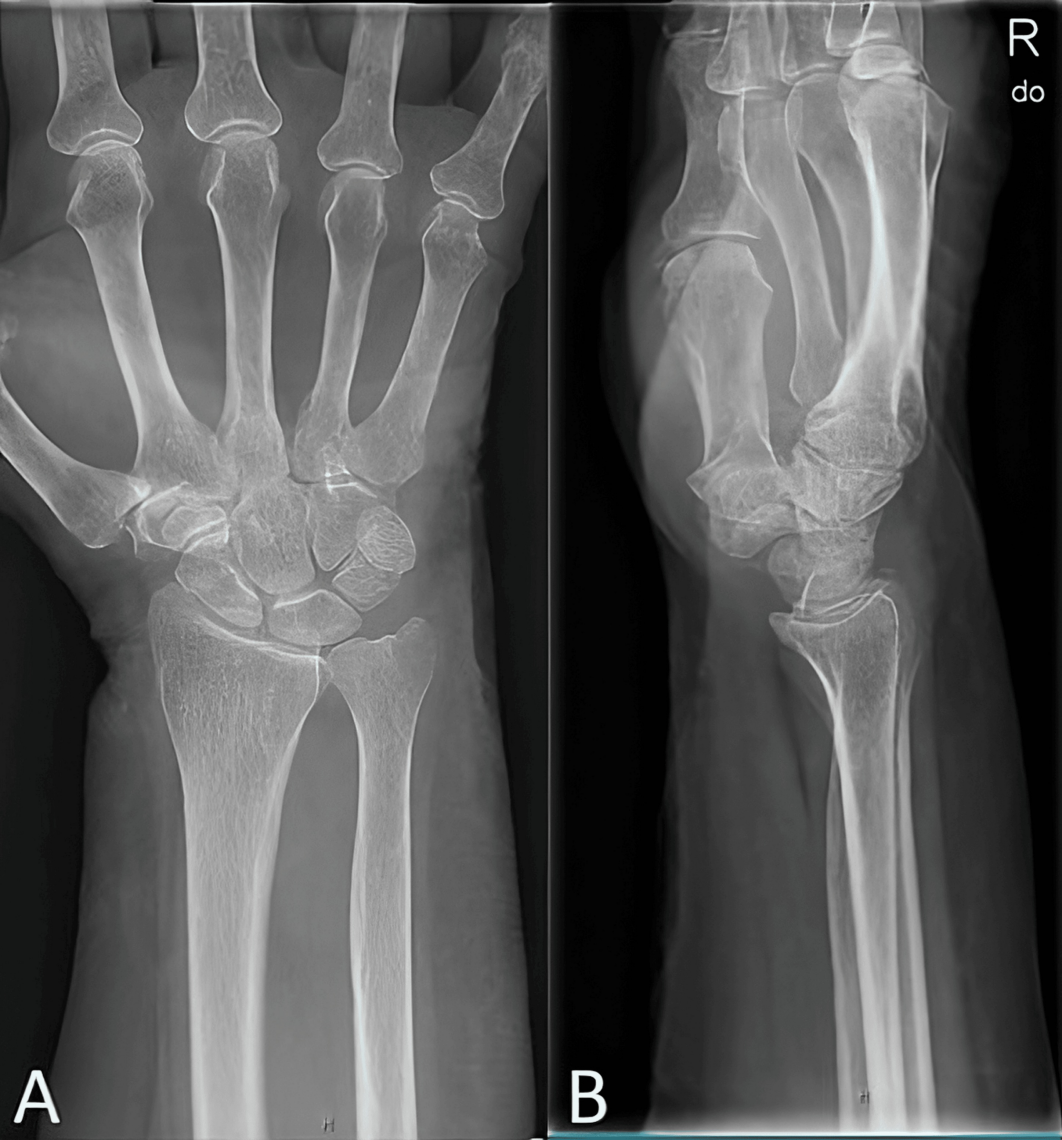
Posteroanterior (A) and lateral (B) radiographs of the right wrist, showing the proper anatomical position of the DRUJ at three weeks after closed reduction. DRUJ: distal radioulnar joint

Correct placement of the joint was confirmed with images acquired using a C-arm image intensifier intraoperatively. A standard longarm cast was then applied. Three weeks later, at the first orthopaedic follow-up, X-rays were obtained again, confirming proper positioning of the radius and ulna at the DRUJ (Figures 4A-4B).

**Figure 5:**
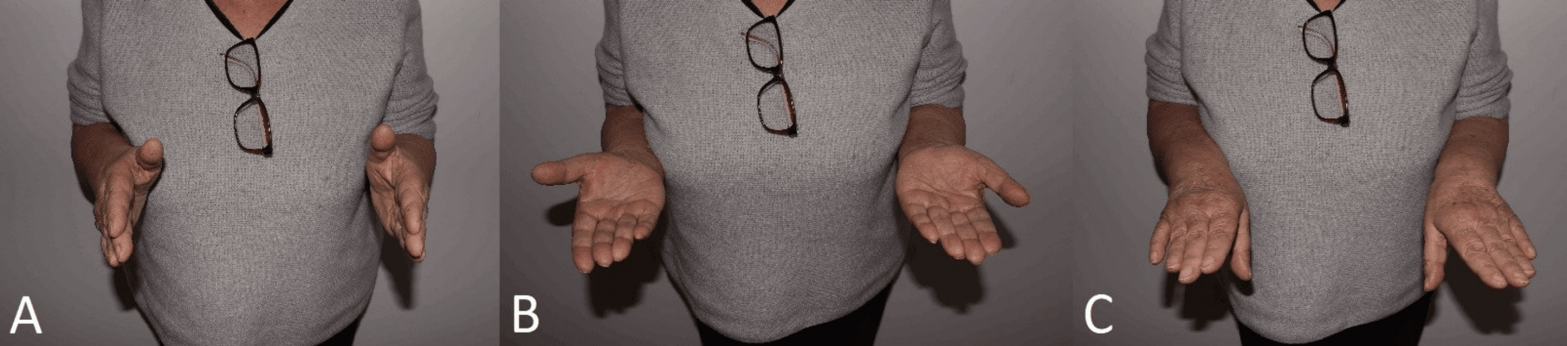
Images at five weeks after injury. Forearms in neutral position (A), supination (B) and pronation (C) showing no limitation in the range of motion.

The longarm cast was removed and switched to a removable forearm splint, which she wore for another two weeks. At this point, an ultrasound of the joint was performed, showing residual joint effusion. The patient reported only slight persistent pain at the wrist on extension and on pronosupination. The radioulnar and dorsovolar range of motion (ROM), as well as supination and pronation, were unrestricted (Figures 5A-5C). The pain did not necessitate analgesics, and she denied limitations in her daily activities. In addition, the patient also received ambulatory physiotherapy.

## Discussion

In this case report, we focused on the biomechanics of DRUJ dislocation, including a brief literature review. The articulation is a pivot joint and is formed by the sigmoid notch of the distal radius and the corresponding surface, the head of the ulna. The two surfaces do not fit perfectly into each other, as the radius of curvature is greater at the sigmoid notch (18 mm) than at the ulnar head (8 mm) [2]. However, great variation in the anatomy occurs amongst different individuals. In the neutral position, these two have the most contact. The degree of contact decreases markedly with pronosupination. Therefore, the DRUJ lacks sufficient bony stability in itself, in contrast to other joints [3]. Instead, the main stability of DRUJ is provided by the triangular fibrocartilage complex (TFCC), which is composed of the palmar and dorsal radioulnar ligaments (RULs), the triangular fibrocartilage disc and ulnocarpal ligaments [4]. It serves as both a force-transmitting and a stabilising structure [5]. The RULs in particular ensure the stability of the DRJU and remain intact during supination and pronation. In a study, Lo et al. found the translation of the ulnar head to be about 10% (~2-4 mm) of the dorsopalmar length of the sigmoid notch [6]. Both RULs consist of superficial and deep fibres, of which the main intrinsic stability is attributed to the latter. They are the most isometric and hence will undergo the least extension when the forearm pronates or supinates. The dorsal deep fibres are tense during supination to prevent ulnar head dislocation in the palmar direction, and palmar fibres will prevent dorsal displacement accordingly [7]. Therefore, the mechanism of injury in volar DRUJ dislocation is usually hypersupination, which brings the ulnar head anterior relative to the radius. As the ulnar head moves volar, the dorsal RUL and the volar joint capsule are disrupted [8].

Even if deep fibres of the RUL are ruptured, sufficient stability can be provided by the remaining structures of the TFCC, such as the superficial fibres of the RUL and the ulnocarpal ligament complex [9,10]. If the deep RULs are severed, palmar translation increases only minimally to 5 mm. The main secondary static support comes then from the distal interosseous membrane (DIOM), mainly from its distal oblique bundle (DOB). Again, considerable anatomical differences exist in the thickness of the DIOM [11,12]. When additionally, the DIOM is impaired, palmar translation (as well as dorsal translation) then increases significantly to 17 mm, as reported by Omokawa et al. [13]. Dynamic secondary support is supplied by the pronator quadratus muscle and the extensor carpi ulnaris (ECU) [14].

A high level of suspicion for associated fractures in DRUJ dislocations is warranted by the physician, as it might have implications regarding the treatment. The diagnosis can easily be missed as the displacement can be small, and even slight rotation on X-ray films can obscure the misalignment of the distal radius and ulna [13]. If uncertainty exists, CT and/or MRI scans should be obtained. If closed reduction can be achieved, the stability of DRUJ can be sufficiently regained after an appropriate healing time with immobilisation in a long-arm cast, which is usually applied for three to six weeks.

We conducted an online literature review using open sources. O'Malley et al. identified 99 cases of volar DRUJ without fractures. They found the patients to be younger (median age: 32 years) and male (63%). About half of the patients could be treated with closed reduction. Although the number of cases was very limited, the findings seemed plausible. The mechanism of injury is often traumatic and occurs more often in younger men because they tend to be involved in higher-risk activities (sports, physical altercations, etc.) [15].

Dorsal dislocation is more common due to the mechanism of injury. DRUJ dislocations are commonly due to falls on an outstretched hand, where the position of the hand is usually in pronation. As described, this leads to diminished joint stability on the dorsal side, causing the ulnar head to dislocate dorsally, rather than in the volar direction. In our patient, we hypothesise that she fell backwards on her supinated hand because she was holding the IV pole by her right side before letting it go.

This case shows that closed reduction and immobilisation can be the proper choice of successful first-line treatment for volar DRUJ dislocation in an older and frail individual. A literature review shows that our patient, aged 88 years, is the oldest known case with this injury. Hand grip strength was not assessed, as she reported no limitations in her daily activities. The full ROM was unrestricted at three months.

A weakness of this case, from a scientific perspective, is that the TFCC was not assessed by MRI. It was not done because the patient did not report any neurological deficits, the repositioning could be achieved successfully and it would not have altered the decision of treatment, additionally, considering her old age. It remains unclear to what extent the RUL or DIOM was injured in her.

## Conclusions

The number of cases with successful closed reduction suggests that this approach is feasible at first. In our view, surgical reduction should be attempted in cases after closed reduction has failed or if there is an accompanying fracture that needs open reduction and internal fixation.
